# What Matters on Rural Left-Behind Children’s Problem Behavior: Family Socioeconomic Status or Perceived Discrimination

**DOI:** 10.3390/ijerph20021334

**Published:** 2023-01-11

**Authors:** Wangqian Fu, Rui Xue, Hongqin Chai, Wenxiang Sun, Fangrui Jiang

**Affiliations:** 1 Faculty of Education, Beijing Normal University, Beijing 100875, China; 2 School of Education, Beijing Sport University, Beijing 100091, China; 3 Social Science Division, University of Chicago, Chicago, IL 60637, USA

**Keywords:** left-behind children, problem behavior, family socioeconomic status, perceived discrimination

## Abstract

With the acceleration of China’s industrialization and urbanization, there is a large number of left-behind children in China who are reported with more challenges in development. The study aims to analyze the differences in family socioeconomic status (SES) and discrimination perception between left-behind children and non-left-behind children and further explore whether SES or discrimination perception has a greater impact on the problem behaviors of left-behind children. We found the family SES of left-behind children was significantly lower than that of non-left-behind children; left-behind children’s perceived discrimination was significantly higher than that of non-left-behind children; perceived discrimination of left-behind children had a greater impact on their problem behavior than the family SES.

## 1. Introduction

With the acceleration of China’s industrialization and urbanization, a large number of rural labor force flows into cities, resulting in the emergence of left-behind children whose parents left for urban employment and higher income. The left-behind children refer to children under 16 years old who are left in their hometown because both or one of their parents is working, doing business, or studying outside and need to be taken care of by other relatives, friends, and teachers [1]. Left-behind children have been reported to be subjects of various social and psychological problems, including depression, anxiety, suicidal idealization, substance use, and delinquency [2]. Being left behind has shown a consistently negative impact on mental health through primary school and middle school [3]. As left-behind children enter their adolescence, a crucial period for self-concept development, their disadvantage in social adaptation becomes even more salient [4].

### 1.1. Family Socioeconomic Status (SES) and Left-Behind Children’s Problem Behavior

Poverty is a fundamental risk factor leading to left-behind adolescents’ negative developmental outcomes. Viewing poverty as a multi-dimensional concept, this study uses SES to measure it. Family SES generally includes parents’ occupation, education level, and family income. Low SES is thus linked to multiple social and psychological mechanisms leading to children’s disadvantage. Aside from the direct economic hardship, stressful living environment, fragile family structure, and restricted parental mental resources are all consequences of low SES that lead to children’s long-term academic failure and behavioral problems [5,6,7] and diminished psychological well-being [8,9,10,11]. It has also been found that the effect of income poverty, subjective felt financial stress, and material hardship can be cumulative, and the detrimental impact of low SES on children’s problem behavior is largest with those factors combined [12,13,14]. Adolescents’ self-esteem has also been found to be diminished by poverty, and the relationship is partly mediated by relational peer bullying, which suggests that poverty cause a loss of power in social interaction and, in turn, affects self-esteem [15,16].

Seeking higher income is the primary motivation for rural parents to migrate, and households with left-behind children are characterized by low parental education levels and monthly income [3,17]. Thus, living in socially and economically disadvantaged rural areas with often unstable family structures and stress caused by inadequate family care performed by often over-aged and low-educated caregivers [18,19], left-behind adolescents are likely to be victims of such cumulative negative effects of low family SES. Indeed, studies on left-behind children in China have shown that due to low family SES, parents lack time, energy, and money to fully participate in children’s lives, and children are thus faced with difficulty in getting the necessary resources for them to succeed, which in turn causes low self-esteem [20,21]. Caregivers’ low SES is significantly associated with adolescents’ emotional and conduct problems [22]. Low family SES has also been found to be associated with non-suicidal self-injury behaviors as well as the risk of suicide mediated by increased peer bullying among left-behind children [23,24].

### 1.2. Discrimination and Left-Behind Children’s Problem Behavior

Discrimination is another salient risk factor for children’s self-esteem and problem behaviors. Two theoretical frameworks supported understanding the association between perceived discrimination with self-esteem and problem behaviors. The symbolic interactionist perspective indicates that perceived negative social evaluation lead adolescents to internalize negative self-image and show a corresponding behavioral reaction. For example, adolescents’ reflected appraisals from parents and peers are significantly associated with self-concept and, in turn, have a salient impact on delinquent behaviors [25]. The social comparison perspective, on the other hand, indicates that the awareness of the unjust treatment received by one’s group, compared to an adjacent reference group, will result in the cognitive construction of an inferior social position and corresponding emotional reaction of resentment, which in turn lead to individual or collective strategies of changing the status quo [26]. The phenomenon of left-behind adolescents is itself a direct result of discriminatory institutional arrangements preventing the rural population from enjoying the social resources in cities [18]. Therefore, it is hardly possible for left-behind adolescents to get rid of their disadvantaged identity, and perceived discrimination may thus result in diminished self-esteem and maladaptive behavioral reactions as a response to the stress caused by unfavorable social comparison and negative self-image.

Dozens of studies found that perceived discrimination was positively associated with problem behaviors in multiple groups of disadvantaged children and adolescents [27,28,29,30,31,32,33]. Taken together, ample empirical evidence suggests that perceived discrimination cause developing minds to form negative self-images and conceptualization of the social environment as hostile to one’s inferior social identity, which in turn led to diminished self-esteem and increased problem behavior. A more recent study found that perceived discrimination against the group identity as migrant children has a significant correlation with the subject’s antisocial behavior as well as self-esteem, which mediated the relationship between discrimination and antisocial behavior [34]. Both quantitative and qualitative data suggested that left-behind children tend to attribute other’s discriminatory attitudes to their parents’ absence [35] and form extreme attacking or avoiding behavioral patterns in social interaction as a response to felt stress [36,37]. Perceived discrimination is associated with children’s problem behavior and limited ability to seek and utilize social support [38,39,40].

### 1.3. The Current Study

There are a large number of left-behind children in China, and more and more studies focus on the development of this group of children, especially what factors influence their problem behaviors. Differences in SES provide more information about environmental inequities [12], while certain complexities exist in this picture. One recent meta-analysis showed that family SES is negatively related to internalized problem behaviors, while the relationship may not be linear-dependent since problem behavior may exist with children enjoying higher SES may exhibit equally heightened problem behaviors [14]. Thus, the exact role of low family SES in left-behind children’s problem behaviors needs further investigation. In addition, the awareness of a “left-behind” identity fueled by discrimination leads to hypersensitivity to adversity in a social environment and results in distorted social functioning in left-behind children [40]. Therefore, it is worthwhile to examine whether perceived discrimination has a more important effect on left-behind children’s problem behavior. The study aims to analyze the differences in SES and discrimination perception between left-behind children and non-left-behind children and further explore whether SES or discrimination perception has a greater impact on the problem behaviors of left-behind children. It will help to understand the causes of problem behaviors of left-behind children, put forward feasible suggestions, promote the social adaptation of left-behind children, and play a certain role in avoiding the occurrence of problem behaviors.

## 2. Method

### 2.1. Participants and Procedures

Voluntary sampling was applied in the study. We contacted principals of elementary and secondary rural schools in Henan province to obtain permission to deliver the questionnaires to students. A total of 9 schools were willing to participate, and 473 students in grades 5–9 took part in the survey. All participants provided informed consent and were told that they were willing to participate and could withdraw at any time. Cattell (1978) believed this ratio of sample size and the number of variable items should be in the range of 3 to 6 [41]. To meet the criterion, the sample size should be more than 300 (with 10% invalid questionnaires in assumption) in the study. A total of 473 questionnaires were distributed, and 473 questionnaires were collected. After removing questionnaires with incomplete repeated answers and missing answers of more than 30%, 438 valid questionnaires were left, and the effective rate was 92.60%. The average age of the participants was 10.48 years old, and the standard deviation was 1.286 years old. There were 284 left-behind children, accounting for 64.8%, and 154 non-left-behind children, accounting for 35.2%. The demographic information of the participants is shown in Table 1.

### 2.2. Measures

#### 2.2.1. Family Socioeconomic Status

Family Socioeconomic Status (family SES) was measured by the education level of parents, parental occupation type, and family monthly income [42,43], which was self-reported by the participants. The educational level of parents was divided into six grades, from “primary school” to “master or above”, with a score of 1–6, respectively. The parents’ occupation type was divided into five grades: “1 = temporary workers, unemployed workers, unskilled and agricultural workers”, “5 = professional senior managers, senior professional and technical personnel, professional supervisors”, with a score of 1–5, respectively. Family monthly income from “less than 1000 RMB” to “more than 30,000 RMB” was assigned a score of 1–6. Finally, the parent’s education level, occupation type, and family monthly income score was converted into standard scores. Then principal component analysis was performed, and the score of family SES was calculated according to the following formula [44]: SES = (*β*_1_ × Z education level of parents +*β*_2_ × Z parental occupation type +*β*_3_ × Z family monthly income)/f. *β*_1_, *β*_2,_ and *β*_3_ were factor loadings, f was the eigenvalue, and the higher the score, the higher the socioeconomic status of the family.

#### 2.2.2. Perceived Discrimination

The perceived discrimination questionnaire for left-behind children developed by Fu et al. (2016) was used [36]. The detailed process of developing the Chinese discrimination perceptions of left-behind children Scale was shown in Fu et al.’s study [45]. The scale consisted of four dimensions: attack (e.g., “Some people express some prejudices about left-behind children in front of me.”), behavior discrimination (e.g., “When I am in difficulty, there will be some problems when I go to relatives or friends of my parents for help“), avoidance (e.g., “because my father or mother is not around, others are not willing to talk to me”), and speech discrimination (e.g., “Some people express some prejudices about left-behind children in front of me.”), with a total of 20 items. The scale was scored on a four-point scale, with 1 = “no” and 4 = “often”. The higher the score, the higher the degree of discrimination experienced by left-behind children. In this study, the internal consistency coefficient of the scale was excellent (α = 0.853), and the fitting index of confirmatory factor analysis (CFA) was good (χ^2^/df = 2.132, GFI = 0.931, AGFI = 0.906, RMSEA = 0.051), representing good reliability and validity of the scale.

#### 2.2.3. Problem Behavior

The adolescent behavior self-assessment questionnaire compiled by Cui was used to measure the problem behavior of the participants [46]. The questionnaire consisted of six dimensions: learning maladaptation (e.g., “being easily distracted or unfocused”), aggressive behavior (e.g., “impulsive or rude behavior, hitting people”), disciplinary violation behavior (e.g., “disobedience, homework copying, smoking”), withdrawals (e.g., “not associating with classmates and always trying to avoid difficulties”), neuroticism (e.g., “sullen, sad, depressed, and feeling that someone is making fun of you”), and exam anxiety (e.g., “not sleeping well the day before the test, and nervous when hearing of the test”), with a total of 60 items. The questionnaire adopted a four-point Likert scale (from 1 = no to 4 = often). The higher the score, the more problem behaviors the subjects had. In this study, the internal consistency coefficient of the scale was excellent (α = 0.957), and the fitting index of CFA was good (χ^2^/df = 2.190, GFI = 0.968, AGFI = 0.960, RMSEA = 0.071), indicating the scale has good reliability and validity.

### 2.3. Statistical Analysis

We used SPSS26.0 software for data statistics and analysis in this study. Firstly, descriptive statistics of demographic information such as gender and grade were carried out. Secondly, the independent-samples *t*-test was used to compare the family SES, perceived discrimination, and problem behavior between left-behind children and non-left-behind children. Pearson correlation analysis was used to explore the correlation between family SES, perceived discrimination, and problem behavior of left-behind children and non-left-behind children. Finally, regression analysis was used to explore the impact of family SES and perceived discrimination on children’s problem behavior, and the magnitude of the impact was compared.

## 3. Result

### 3.1. Family SES with Left-Behind and Non-Left-Behind Children

The participants were divided into left-behind children and non-left-behind children, and the family SES of the two groups was compared by *t*-test. It could be seen that, in general, the family SES of left-behind children (M = 7.59, SD = 2.16) was significantly lower than that of non-left-behind children (M = 8.31, SD = 2.42) (t = −3.189, *p* < 0.01).

### 3.2. Comparison of Perceived Discrimination and Problem Behavior between Left-Behind and Non-Left-Behind Children

The perceived discrimination of left-behind children and non-left-behind children was compared by *t*-test. In general, left-behind children’s perceived discrimination was significantly higher than that of non-left-behind children (t = 2.879, *p* < 0.01).

The problem behavior and its subscales of left-behind children and non-left-behind children were compared by multivariate analysis, and the results are shown in Table 2.

As shown in Table 2, there was no significant difference in problem behavior between left-behind children and non-left-behind children. However, the comparative analysis of all dimensions of problem behaviors between left-behind children and non-left-behind children showed that left-behind children had significantly more disciplinary violation behaviors than non-left-behind children (F = 4.873, *p* < 0.05), while there was no significant difference in learning maladaptation, aggressive behavior, withdrawals, neuroticism and exam anxiety.

### 3.3. Relationships between Family SES, Perceived Discrimination, and Problem Behavior of Left-Behind and Non-Left-Behind Children

Pearson correlation analysis was conducted on family SES, perceived discrimination, and problem behavior of left-behind children and non-left-behind children, respectively, and the results are shown in Table 3.

It could be seen that there was no significant correlation between family SES and perceived discrimination in different groups of children, while perceived discrimination was significantly positively correlated with problem behavior in both groups (r = 0.333, *p* < 0.01; r = 0.212, *p* < 0.01). The family SES of left-behind children was negatively correlated with problem behavior (r = −0.124, *p* < 0.05). However, there was no significant correlation between family SES and problem behavior of non-left-behind children.

### 3.4. Association of Family SES and Perceived Discrimination with Problem Behavior of Left-Behind and Non-Left-Behind Children

To further explore the influence of family SES and perceived discrimination on children’s problem behavior, the gender, age, and grade of the subjects were controlled, and the regression analysis was conducted with family SES and perceived discrimination as independent variables and problem behavior as a dependent variable, respectively. The results are shown in Table 4.

It could be seen that the family SES of left-behind children and non-left-behind children had no significant association with problem behavior. The perceived discrimination of both groups had a significant positive association with problem behavior (*β =* 0.335, *p* < 0.001; *β* = 0.211, *p* < 0.01). The standardized regression coefficient can be used to compare the relationships between different independent variables on dependent variables. The larger the coefficient value is, the greater the effect will be [47]. The regression equation standardization coefficient of perceived discrimination with problem behavior was higher, indicating that the perceived discrimination of left-behind children had a greater association with their problem behavior than the family SES.

## 4. Discussion

### 4.1. What Does a Lower Family SES Mean for Children

The results of this study showed that the family SES of left-behind children was significantly lower than that of non-left-behind children, which was consistent with the results of previous studies [44]. When the family’s SES was low, and the local salary could not meet the needs of the family, the parents needed to go out to work to support the family. When parents go out to work, they have to control their expenses by eating and living together with their colleagues and leaving their children at home could reduce the cost of renting, which resulted in the “left-behind” situation of their children [48].

### 4.2. Left-Behind Children Have Higher Perceived Discrimination and Disciplinary Violation

The results of this study showed that left-behind children’s perceived discrimination and disciplinary violation were significantly higher than those of non-left-behind children, which was consistent with the results of relevant studies [49,50,51]. Compared with children in normal situations, left-behind children certainly affected their psychological development and externalizing behaviors, making their psychological development relatively poor and showing more external problem behaviors. First of all, the higher perceived discrimination of left-behind children could be caused by two factors. First, affected by parent-child separation, left-behind children are often very insecure [52], and the lack of security would make left-behind children increase their awareness of aggressive behaviors (such as fear of being bullied by others). Second, left-behind children were “stigmatized”. Studies have pointed out that school administrators, teachers, and the people who lived beside left-behind children, as well as some researchers, exaggerate the remaining negative effect; left-behind children “stigmatized” tended to be more apparent, which could lead to a companion of left-behind children bias, treated unfairly, which caused the “stigmatized” left-behind children had a higher perception of discrimination [53]. Secondly, in terms of disciplinary violation, the parents of left-behind children worked abroad for a long time. They lacked care for their children, which was an important factor for left-behind children’s disciplinary behaviors. Empirical studies have pointed out that parental warmth could negatively predict children’s disciplinary behaviors. The higher the level of warmth, the fewer children’s disciplinary behaviors [54]. Moreover, insufficient warmth to children would make them feel more negative emotions, which were specifically manifested in sensitivity, inferiority, anxiety, depression, etc. [55,56]. Depression was considered to be the cause of disciplinary violation behavior by the acting out theory [57]. The Coercion Model also suggested that poor parenting, such as lack of love and discipline, reinforced the Coercion behavior in early adolescence, leading to more Coercion [58]. Left-behind children might hope to attract the attention of their parents through their disciplinary violation behaviors so that they could get more care and attention.

### 4.3. The Impact of Perceived Discrimination on Left-Behind Children Was More Noteworthy Than That of Family SES

In this study, problem behavior was taken as the dependent variable to explore the predictive effects of family SES and perceived discrimination. The results showed that perceived discrimination had a significant predictive effect on problem behavior, and perceived discrimination of left-behind children has a greater impact on their problem behavior than family SES.

Firstly, this study found that there was no significant correlation between children’s family SES and perceived discrimination. It was consistent with the research results of Romero and Roberts [59]. When the economic level reaches a certain level, the perceived discrimination caused by family SES decreases, and the correlation between them also decreases.

Secondly, in the group of left-behind children, there was a significant negative correlation between family SES and problem behavior, which was consistent with the results of previous studies [60,61,62,63]. The lower the family SES, the more problem behavior children had. According to the Ecological System Theory proposed by Bronfenbrenner, individual development was nested in a series of interacting environmental systems. As one of the main environments for children’s development, the family has an important influence on individual development. The education level and occupation level of parents of left-behind children were generally low and left-behind children were exposed to a higher risk of family SES. Left-behind children living in low SES lack supervision from their parents. Supervision from grandparents or other guardians was mostly limited to the living level, and the lack of favorable supervision of children’s learning and behavior and effective psychological guidance led to social adaptation problems for left-behind children [64]. Externalizing behavior was more likely to show learning maladaptation, disciplinary violation behavior, aggressive behavior, and other problems.

In addition, this study found that perceived discrimination had a greater impact on problem behaviors than family SES; that was, the impact of perceived discrimination on problem behaviors of left-behind children was more noteworthy than family SES. Low family SES to a certain extent, certainly made the development of the children a certain risk, but this did not have the inevitability of risk, its action mechanism tended to adjust by other factors, such as parents’ upbringing, community environment, and so on [65], and this risk was primarily a children’s psychological change of consciousness, such as higher levels of depression, loneliness, social anxiety, etc. [66], which did not directly lead to problem behavior in children. The generation of children’s problem behavior was mainly due to the accumulation of various risk factors, which strengthened the adverse effect of another risk factor and led to children’s negative psychological problems. These risk factors included various stressful life events, traumatic events, and the accumulation of pressure on the individual and the environment [67]. Left-behind children had higher perceived discrimination, which led to a long-term stressful experience. Negative external stimuli were more likely to turn into risk factors and accumulate continuously, eventually leading to problem behavior of left-behind children. It was difficult for educators to change the family SES of left-behind children so as to reduce their negative effects. However, they could take positive measures from the aspects of educational concepts and methods to reduce the perceived discrimination of left-behind children and pay attention to the healthy psychological development of left-behind children so as to reduce the occurrence of problem behavior.

## 5. Conclusions

The study analyzed the association of SES and discrimination perception and problem behavior for rural left-behind children and non-left-behind children in China. Thes study found that there was no significant correlation between family SES and discrimination perception in different groups of children, while discrimination perception was significantly positively correlated with problem behavior in both groups. The family SES of left-behind children was negatively correlated with problem behavior. However, there was no significant correlation between family SES and problem behavior of non-left-behind children. The family SES of left-behind children and non-left-behind children had no significant association with problem behavior. The discrimination perception of both groups had a significant positive association with problem behavior. Regression analysis found the discrimination perception of left-behind children had a higher association with their problem behavior than the family SES. Therefore, educators should take strategies to alleviate left-behind children’s discrimination perception, which is helpful to reduce their problem behaviors.

## Figures and Tables

**Table 1:** Demographic information of the participants. (*N* = 438).

Students’ Information	*N*	100%
Gender		
Male	219	50.0
Female	219	50.0
Grade		
Grade 5~6	100	22.8
Grade 7	112	25.6
Grade 8	121	27.6
Grade 9	125	24.0
Left-behind or not		
Yes	284	64.8
No	154	35.2
Situation of parents		
No one works outside	145	33.1
Both work outside	115	26.3
Father works outside	165	37.7
Mother works outside	13	3.0
Time of parents’ working outside		
No one works outside	145	33.1
Less than 6 months	72	16.4
6 months to 1 year	117	26.7
1–2 years	39	8.9
2–4 years	21	4.8
More than 5 years	44	10.0

**Table 2:** Comparison of Problem Behaviors between Left-Behind and Non-Left-Behind Children.

	Left-Behind (*N* = 284)		Non-Left-Behind (*N* = 154)		F
	M	SD	M	SD	
Learning Maladaptation	1.78	0.61	1.74	0.63	0.474
Aggressive Behavior	1.33	0.43	1.31	0.44	0.363
Disciplinary Violation	1.17	0.35	1.10	0.19	4.873 *
Withdrawals	1.38	0.47	1.30	0.41	3.489
Neuroticism	1.35	0.51	1.33	0.51	0.167
Exam Anxiety	2.01	0.89	2.07	0.92	0.480
Problem behaviors	1.43	0.38	1.39	0.34	1.348

Note: *: *p* < 0.05. M = Mean, SD = Standard Deviation.

**Table 3:** Pearson correlation analysis of family SES, perceived discrimination, and problem behavior between left-behind children and non-left-behind children.

	Family SES	Perceived Discrimination	Problem Behavior
Family SES	1		
Perceived Discrimination	−0.113 ^a^ (−0.095 ^b^)	1	
Problem Behavior	−0.124 ^a^* (−0.078 ^b^)	0.333 ^a^**(0.212 ^b^**)	1

Note: *: *p* < 0.05, **: *p* < 0.01; ^a^: left-behind children, ^b^: non-left-behind children.

**Table 4:** Effects of family SES and perceived discrimination on problem behavior between left-behind and non-left-behind children.

Predictor Variable	Problem Behavior	
	Standardizeβ	ΔR^2^
Family SES	−0.111 ^a^ (−0.058 ^b^)	0.011 ^a^ (0.003 ^b^)
Perceived Discrimination	0.335 ^a^*** (0.211 ^b^**)	0.107 ^a^ (0.043 ^b^)

Note: **: *p* < 0.01, ***: *p* < 0.001; ^a^: left-behind children, ^b^: non-left-behind children.

## Data Availability

The datasets generated and analyzed during the current study are available from the corresponding author on reasonable request.
